# Supplementary material for: Saccharomyces boulardii (CNCM I-745) improves intestinal damage in sepsis by remodeling intestinal flora structure
Source: Front Cell Infect Microbiol. 2025 Jul 31;15:1602792. doi: 10.3389/fcimb.2025.1602792 (PMC12350329; doi:10.3389/fcimb.2025.1602792)
Supplement: Supplementary file 1 [file DataSheet1.zip › summary/summary/7_Differential_analysis/2_LEfSe_result/Group_AW4_vs_AW0_0.05_3.0/LEfSe_Group_AW4_vs_AW0.s.barplot.pdf]

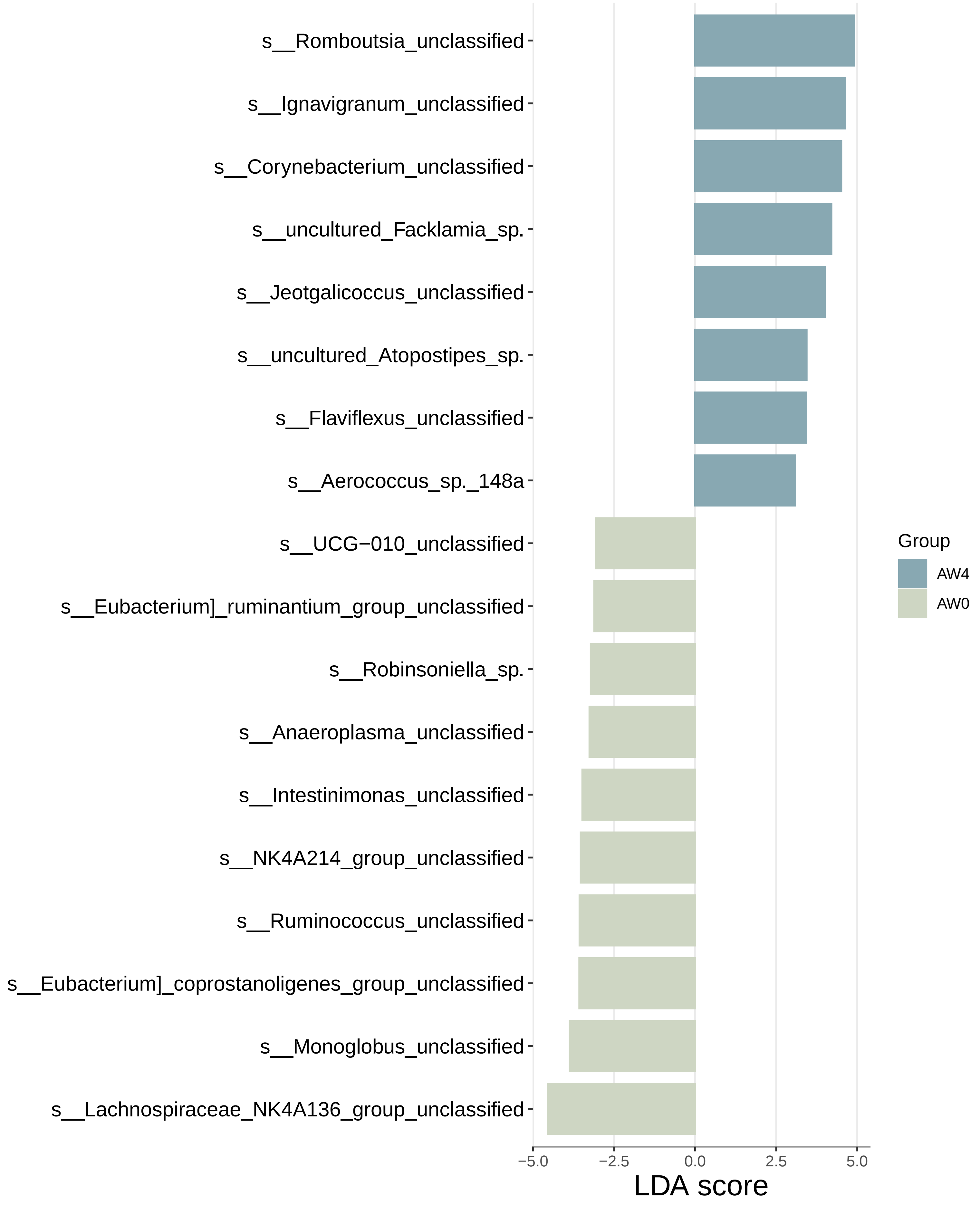
s__Romboutsia_unclassified
s__Ignavigranum_unclassified
s__Corynebacterium_unclassified
s__uncultured_Facklamia_sp.
s__Jeotgalicoccus_unclassified
s__uncultured_Atopostipes_sp.
s__Flaviflexus_unclassified
s__Aerococcus_sp._148a
s__UCG−010_unclassified
s__Eubacterium]_ruminantium_group_unclassified
s__Robinsoniella_sp.
s__Anaeroplasma_unclassified
s__Intestinimonas_unclassified
s__NK4A214_group_unclassified
s__Ruminococcus_unclassified
s__Eubacterium]_coprostanoligenes_group_unclassified
s__Monoglobus_unclassified
s__Lachnospiraceae_NK4A136_group_unclassified
−5.0
−2.5
0.0
2.5
5.0
LDA score
Group
AW4
AW0
